# Computational Investigation of Precursor Blocking during Area-Selective Atomic Layer Deposition Using Aniline as a Small-Molecule Inhibitor

**DOI:** 10.1021/acs.langmuir.2c03214

**Published:** 2023-03-15

**Authors:** I. Tezsevin, J. F. W. Maas, M. J. M. Merkx, R. Lengers, W. M. M. Kessels, T. E. Sandoval, A. J. M. Mackus

**Affiliations:** † Department of Applied Physics, Eindhoven University of Technology, Post Office Box 513, 5600 MB Eindhoven, Netherlands; ‡ Department of Chemical and Environmental Engineering, Universidad Técnica Federico Santa Mariá, Santiago 2340000, Chile

## Abstract

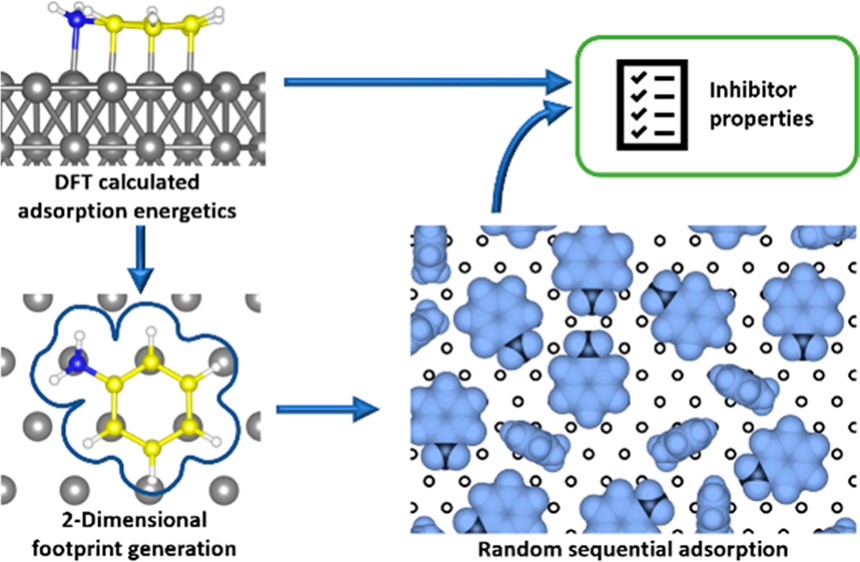

Area-selective atomic layer deposition using small-molecule inhibitors (SMIs) involves vapor-phase dosing of inhibitor molecules, resulting in an industry-compatible approach. However, the identification of suitable SMIs that yield a high selectivity remains a challenging task. Recently, aniline (C_6_H_5_NH_2_) was shown to be an effective SMI during the area-selective deposition (ASD) of TiN, giving 6 nm of selective growth on SiO_2_ in the presence of Ru and Co non-growth areas. In this work, using density functional theory (DFT) and random sequential adsorption (RSA) simulations, we investigated how aniline can effectively block precursor adsorption on specific areas. Our DFT calculations confirmed that aniline selectively adsorbs on Ru and Co non-growth areas, whereas its adsorption on the SiO_2_ growth area is limited to physisorption. DFT reveals two stable adsorption configurations of aniline on the metal surfaces. Further calculations on the aniline-functionalized surfaces show that the aniline inhibitor significantly reduces the interaction of Ti precursor, tetrakis(dimethylamino)titanium, with the non-growth area. In addition, RSA simulations showed that the co-presence of two stable adsorption configurations allows for a high surface inhibitor coverage on both Co and Ru surfaces. As the surface saturates, there is a transition from the thermodynamically most favorable adsorption configuration to the sterically most favorable adsorption configuration, which results in a sufficiently dense inhibition layer, such that an incoming precursor molecule cannot fit in between the adsorbed precursor molecules. We also found that, as a result of the catalytic activity of the metallic non-growth area, further reactions of inhibitor molecules, such as hydrogenolysis, can play a role in precursor blocking.

## Introduction

The continued downscaling of feature sizes in integrated circuits leads to new challenges in the semiconductor industry.^1^ Most of the conventional fabrication processes are designed on the basis of the top-down approach, comprising numerous deposition, lithography, and etching steps.^2,3^ At small feature sizes, traditional top-down approaches suffer from alignment issues between the patterning steps.^4^ Therefore, processes exploiting the properties of the underlying pattern instead of purely relying on metrology and patterning precision are needed.^5,6^ During the past decade, area-selective deposition (ASD), a bottom-up alternative enabling self-aligned fabrication schemes, has gained considerable attention in academia and industry.^7−9^ ASD refers to processes that deposit the target material on the areas where the growth is desired (i.e., growth areas), while deposition is avoided on the neighboring areas where the growth is not desired (non-growth areas). The selectivity of deposition on the growth areas can be achieved by exploiting differences in the chemical or physical properties, such as different material compositions, surface terminations, or lattice properties.^6,7,10,11^

In some specific cases, an inherent selectivity may be observed as a result of the nature of the growth and non-growth areas.^8,12^ However, typically, selectivity is achieved via the deactivation of the non-growth area using inhibitors, for example, using self-assembled monolayers (SAMs) or small-molecule inhibitors (SMIs).^6,11−18^ SAMs, containing long-chain hydrocarbons, have conventionally been used as inhibitors to achieve area-selective atomic layer deposition (ALD).^19,20^ However, as a result of the low vapor pressure of these large molecules, application of SAMs typically relies on wet chemistry and requires long reaction times to achieve close packing.^21−24^ These properties limit the compatibility of SAMs with high-volume applications in the industry.^16^ As an alternative, the use of SMIs recently showed promising results for area-selective ALD.^3,15,25−27^ SMIs can be supplied into the vacuum reactor during the deposition via vapor-phase dosing.^28^ As a result, inhibitors can be applied in much shorter time scales in the same vacuum vessel as used for the ALD process and can therefore more easily be integrated in industrial fabrication schemes.^22^ The area-selective ALD approach relying on vapor-phase reapplication of SMIs at the start of every ALD cycle can be employed using both thermal or plasma-enhanced conditions.^22^

To successfully block the interaction of the incoming precursor molecules with the non-growth area during ASD, candidate SMIs should fulfill the following requirements: (i) SMIs should strongly and selectively bind to the non-growth area, and (ii) the SMIs should have a sufficiently high surface coverage on the non-growth area. The selective adsorption of the candidate SMI molecules can be investigated using density functional theory (DFT) calculations on the target growth and non-growth areas. Thus, important information on the adsorption energetics and the adsorption configurations of the SMIs can be obtained. Random sequential adsorption (RSA) simulations^29,30^ can be used to estimate the coverage and packing of inhibitor molecules as extensively discussed in our earlier work.^31^ As a consequence of vapor-phase dosing, SMIs arrive one by one on random surface sites such that close packing of inhibitor molecules cannot be achieved.^9^ This could be compensated by the selection of inhibitor molecules with high enough surface coverage and dense packing to block the incoming precursor molecules. In addition, the precursor blocking performance of the inhibitor can be assessed using RSA simulations as discussed in the Results and Discussion of this manuscript.

Although the use of SMIs for area-selective ALD is a very promising approach, finding suitable SMIs remains a challenging task. In our previous works, acetylacetone (Hacac, C_5_H_8_O_2_) was demonstrated as an inhibitor during the area-selective deposition of SiO_2_ using bis(diethylamino)silane (BDEAS) as the precursor.^26,32^ It was found that the use of the Hacac inhibitor can delay SiO_2_ growth on Al_2_O_3_ non-growth areas up to 20 cycles, corresponding to about 1.5 Å selective SiO_2_ deposition on SiO_2_ growth areas.^26^ Both experiments and DFT calculations showed that Hacac molecules adsorb on an Al_2_O_3_ non-growth area in a mixture of two bonding configurations (i.e., chelate and monodentate), whereas they cannot adsorb to the SiO_2_ growth area. Among these two adsorption configurations, the chelate configuration was found to block BDEAS molecules successfully. However, Hacac molecules adsorbed in monodentate configuration can desorb during the purging step of the experiments or were displaced during the precursor dose and caused the loss of selectivity.^32^ According to these findings, it is needed to investigate all possible adsorption configurations of inhibitor molecules and their effect on the inhibition layer stability. In our recent experimental work, aniline showed promising performance as an inhibitor during the area-selective deposition of TiN on dielectrics as the growth area with respect to metals as the non-growth area. Using aniline as an inhibitor and tetrakis(dimethylamino)titanium (TDMAT) as a Ti precursor, TiN growth was delayed for up to 100 cycles on Co and for more than 200 cycles on Ru non-growth areas. As a result, about 6 nm of selective TiN deposition on SiO_2_ and Al_2_O_3_ was achieved.^28^

**Figure 1:**
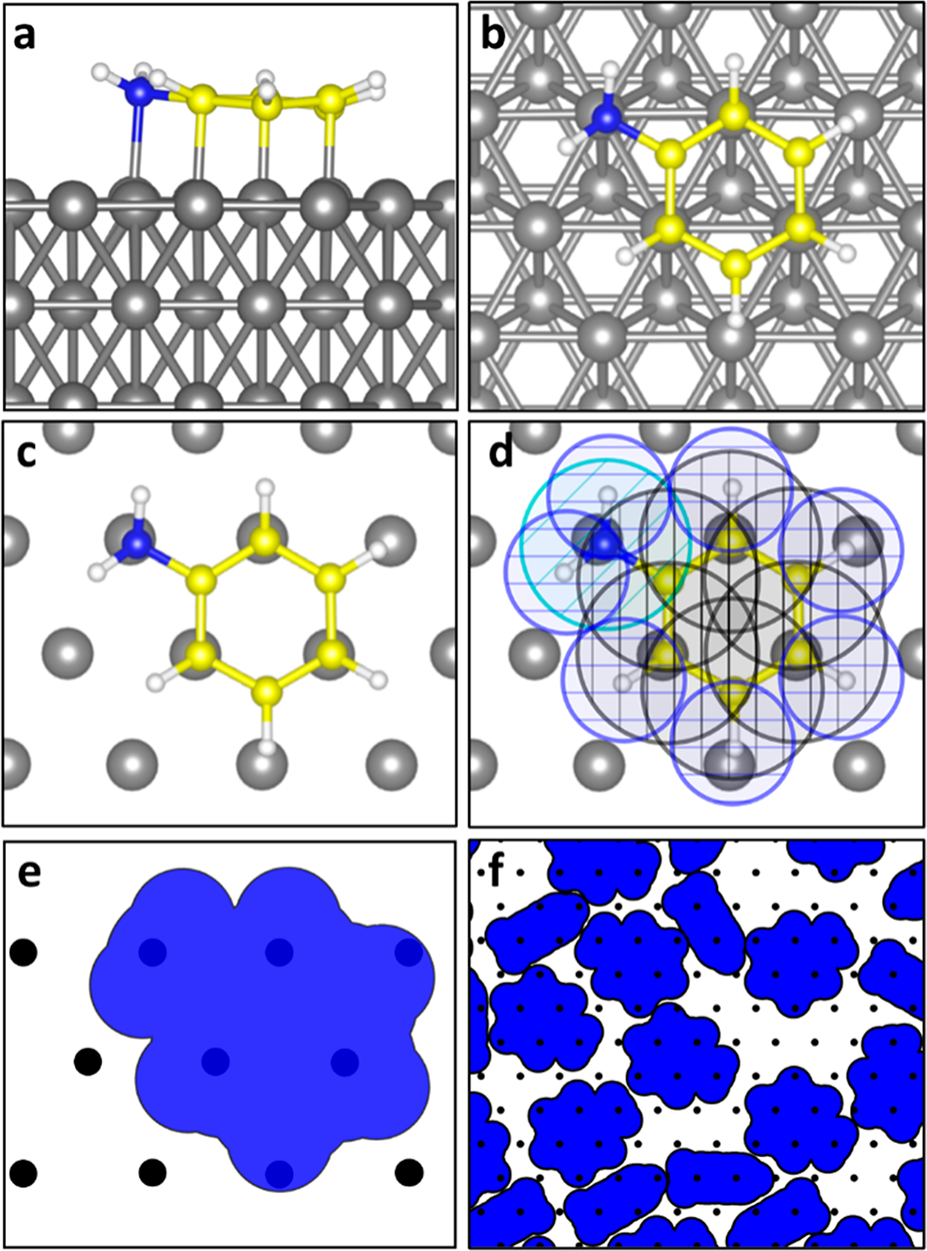
Example of a DFT-calculated adsorption configuration of an aniline molecule shown in the (a) side view and (b and c) top view (gray, surface metal atoms; yellow, carbon; white, hydrogen; and blue, nitrogen). (c) Top layer metal atoms have been used to form the surface model. (d) Disks representing N, C, and H atoms have been placed on the corresponding coordinates for aniline based on their van der Waals radius. (e) Final 2D aniline footprint as input for the RSA simulations has been created as the combination of the disks into a single shape (a union). (f) Section of the substrate with adsorbed molecules as output of the RSA simulation.

In the production of nanoelectronics, various stages involve a substrate that has been patterned with metal and dielectric materials. In this work, the general case of having a metal as the non-growth area is considered. We performed DFT calculations and RSA simulations to investigate the key properties making aniline an effective SMI on metal substrates. DFT calculations were carried out to study the adsorption configurations and stability of aniline molecules on SiO_2_ representing the dielectric growth area and on Ru and Co surfaces representing the metal non-growth areas of a patterned substrate. Considering the catalytic nature of the Ru and Co surfaces, possible hydrogenolysis of aniline on these surfaces was also included in the study. In addition, the DFT results were used as input for the RSA simulations, as illustrated in Figure 1, by modeling the two-dimensional (2D) substrate and the footprint of the adsorbate molecules. Using RSA simulations, we investigated the surface coverage and packing of the inhibitor molecules on the non-growth areas. The outcomes of the DFT calculations and RSA simulations were combined to unveil how aniline effectively blocks precursor adsorption on the non-growth area. Our results reveal that aniline molecules adsorb on the non-growth area in a mixture of strongly bonded configurations. This mixture helps aniline achieve a high surface coverage, resulting in effective precursor blocking.

## Computational Methods

### DFT

The Vienna *Ab Initio* Simulation Package (VASP) was used to perform all DFT calculations reported in this study.^33−35^ The projector augmented wave (PAW) method was employed to describe electron–ion interactions.^36,37^ A kinetic energy cutoff of 400 eV was used for the plane-wave basis set. Calculations were performed on the basis of the Perdew–Burke–Ernzerhof (PBE) exchange–correlation functional of the generalized gradient approximation (GGA), with the dispersion correction D3 and the Becke–Johnson (BJ) damping function.^38−40^ The convergence criteria for structural optimizations were set such that the total forces acting on each atom must be smaller than 0.01 eV/Å. The convergence criterion for the self-consistent field cycle was set to 10^–5^ eV. The Brillouin zone of crystalline Ru and Co metal bulk was integrated using an automatically generated Γ-centered 11 × 11 × 11 *k*-point mesh (11 × 11 × 9 for SiO_2_), whereas a Γ-centered 2 × 2 × 1 mesh was used for the surface calculations.^41^ Gaussian smearing of 0.01 eV was used throughout the study. Using these parameters, optimized lattice parameters of the Ru bulk with *P*63/*mmc* space group (space group number = 194) were found to be *a* = 2.70 Å and *c* = 4.27 Å, which are in a good aggreement with the experimental vaues of 2.71 and 4.28 Å, respectively.^42^ Optimized lattice parameters of the Co bulk with the same space group number were calculated as *a* = 2.46 Å and *c* = 3.99 Å and were also in a good aggreement with the experimental values of 2.50 and 4.06 Å, respectively.^43^ The lattice parameters of the α-quartz phase of SiO_2_ with *P*3121 space group (space group number = 152) were calculated as *a* = 4.80 Å and *c* = 5.32 Å, which were also in line with the experimental values of *a* = 4.91 Å and *c* = 5.40 Å.^44^

Ru(0001) and Co(0001) surface slabs used for the surface calculations were modeled using a four-layer 4 × 4 supercell of the cleaved optimized bulk structure. The metal atoms in the bottom two layers of the surface slab were kept frozen at their bulk positions during adsorption studies. Also, for the SiO_2_(0001) surface, a four-layer (SiO_2_ layers) surface was modeled as a 3 × 3 supercell of the cleaved optimized SiO_2_ bulk structure. As in metal surfaces, the bottom two (SiO_2_) layers were kept frozen at their bulk positions. In line with the previous ALD studies, the top layer of the SiO_2_(0001) surface is fully hydroxylated.^26,45,46^ Periodicity of the slab in the direction perpendicular to the metal surface was avoided by adding a vacuum spacing of 17 Å. At least 10 different starting configurations were studied for the aniline adsorption on metal surfaces. In addition, by keeping the *x* and *y* coordinates the same, the effect of the starting distance of the aniline molecule to the surface was also tested for 2 and 3 Å, to locate possible physisorbed geometries. All of the adsorption configurations reported for aniline were found to proceed directly from the gas phase. In other words, no local maximas or activated pathways were observed in the optimization calculations for aniline adsorption on the metal surfaces. This can be attributed to the reactive nature of metal surfaces. All resulting unique configurations are reported here. The aniline adsorption energies were calculated using
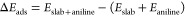
1where *E*_slab+aniline_ is the total energy of the Ru(0001) or Co(0001) slab with adsorbed aniline at its lowest energy position on the metal surface, *E*_slab_ is the total energy of a clean slab, and *E*_aniline_ is the total energy of the aniline molecule. The same procedure is followed for the adsorption of the TDMAT precursor. The energies of the aniline and TDMAT molecules were computed via spin-relaxed calculations in 20 Å cubic cells at the gamma point.

The hydrogenolysis energy was also calculated using a formula similar to eq 1. In this case, the total energy before the hydrogenolysis reaction was subtracted from the total energy of the end products as shown in
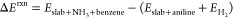
2where *E*_slab+NH_3_+benzene_ is the total energy of formed ammonia and benzene on Ru(0001) or Co(0001) slab, *E*_slab+aniline_ is the total energy of Ru(0001) or Co(0001) slab with adsorbed aniline, and *E*_H_2__ is the energy of a gas-phase H_2_ molecule. Unlike the adsorption process explained above, the hydrogenolysis reaction is not expected to proceed spontaneously as a result of the bond cleavage and formation of the new bonds. The kinetic analysis of this process is left for future mechanistic studies.

### RSA

A lattice RSA algorithm (see Figure S1 of the Supporting Information) developed on the basis of our earlier work has been adopted in this study.^31^ This RSA algorithm simulates one-by-one adsorption of inhibitor molecules on randomly selected sites on a substrate representing the non-growth area during area-selective ALD. The DFT-optimized lattice dimensions of Ru(0001) and Co(0001) surfaces were used to model the substrates with periodic boundary conditions. Both model substrates were prepared to include 5000 adsorption sites, resulting in surface areas of 318 and 273 nm^2^ for the Ru and Co surfaces, respectively. The top-down 2D projections, i.e., 2D footprints, of aniline and benzene molecules were modeled on the basis of the DFT-optimized adsorption configurations of molecules on the metal non-growth areas (see Figure 1 for the procedure and Figure S2 of the Supporting Information for other 2D footprint models). The horizontal aniline configuration is strongly adsorbed to the surface via the interactions of its multiple atoms with the surface, preventing free rotation. Therefore, rotation attempts for the footprint representing the horizontal configuration were considered on the basis of the 6-fold symmetry of the lattice (rotational attempts of 60°). On the other hand, the vertical aniline configuration is attached to the surface via a single bond between N and the surface metal. Hence, it is assumed to be able to rotate freely around the M–N bond. During the RSA iterations, the vertical aniline model was first tested on the basis of the 6-fold symmetry (60° rotation) in random order. Then, if it still did not fit, 1° rotation steps were tested to cover a larger range of possible rotations. A reactive adsorption mode is also included in the algorithm to be able to simulate aniline adsorption with hydrogenolysis upon adsorption. When using the reactive adsorption mode, aniline molecules adsorbed in horizontal configuration were converted to benzene. All RSA results reported in this study were averaged over 20 simulations.

The RSA simulations were used to analyze the inhibitor coverage and surface density. In addition, the gaps in the inhibitor layer (i.e., space not sterically covered by aniline) can be analyzed in terms of their density and size. The effective size of these gaps has been calculated by determining the radius of the largest circular molecule that can be adsorbed on the unoccupied adsorption sites without an overlap with the adsorbed inhibitor molecules (see Figure S3 of the Supporting Information). Please also see ref (31) for more detailed information about the RSA methodology.

## Results and Discussion

**Figure 2:**
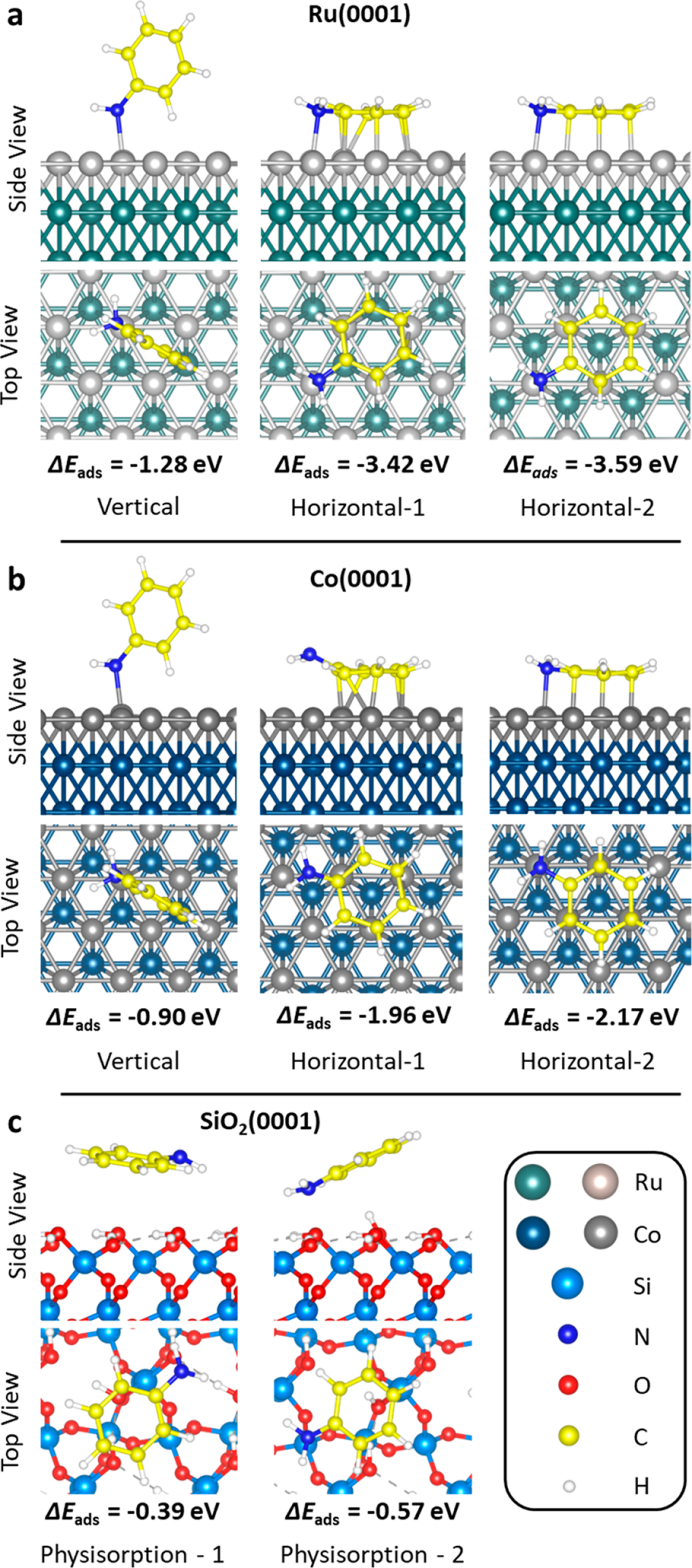
DFT-optimized aniline adsorption configurations and energies on (a) Ru(0001) and (b) Co(0001) surfaces representing the non-growth areas and (c) SiO_2_(0001) surface representing the growth area. The top layer metals are represented in a different color for better top view visualization.

Selective adsorption of the inhibitor molecules on the non-growth area is an essential step to achieve area-selective ALD using SMIs. Therefore, we first investigated the adsorption mechanism of aniline on the Ru(0001) and Co(0001) surfaces representing the non-growth areas and on the SiO_2_(0001) surface representing the growth area. The aniline molecule strongly adsorbs with adsorption energies of −3.59 eV on Ru and −2.17 eV on Co. Three unique thermodynamically favorable adsorption configurations for aniline on Ru and Co were found from DFT, as shown in Figure 2. The aniline configuration with the most favorable adsorption energy on the metal surfaces involves the interaction of the carbon atoms of the aniline aromatic ring and the surface metal atoms, resulting in a “horizontal” adsorption mode. Moreover, there is also a “vertical” configuration for which aniline adsorbs on the surface only through interaction between nitrogen of the amine group and the surface metal, resulting in weaker adsorption than the horizontal configuration. On the basis of these results, it is expected that aniline adsorbs on the metal surfaces through a combination of these horizontal and vertical adsorption configurations while maximizing the horizontal configuration, i.e., the most exothermic adsorption configuration.^47−49^

On the SiO_2_(0001) surface representing the growth area, two unique adsorption configurations with considerably weaker adsorption energies were found. Aniline molecules adsorb on the SiO_2_(0001) surface only via physical interactions between the molecule and the surface. Even in the most favorable adsorption configuration on SiO_2_, the adsorption energy is only −0.57 eV. Molecules adsorbed in these two weak physisorbed adsorption configurations are likely to desorb in the experimental conditions and, thereby, not take part in precursor blocking. The difference between adsorption energetics across surfaces indicates that aniline can selectively adsorb on the metallic non-growth areas, Co and Ru, but not on the SiO_2_ growth area, which is also in agreement with previous experimental results.^28^

These DFT analyses reveal a potential reason for why aniline results in better precursor inhibition performance compared to, for example, Hacac,^32^ for which the presence of the mixture of different adsorption configurations is causing selectivity loss. For the case of aniline, both the horizontal and vertical adsorption configurations bind strongly on the non-growth area and are not expected to desorb during the purging step. In addition, while the weaker (monodentate) configuration of Hacac could provide a pathway for precursor adsorption as a result of its exposed OH group, the phenyl group of the vertical aniline molecules does not have an affinity toward the precursor molecules. The phenyl group can also shield the interaction of the (relatively more reactive) amine group of aniline with the precursor molecules.

**Figure 3:**
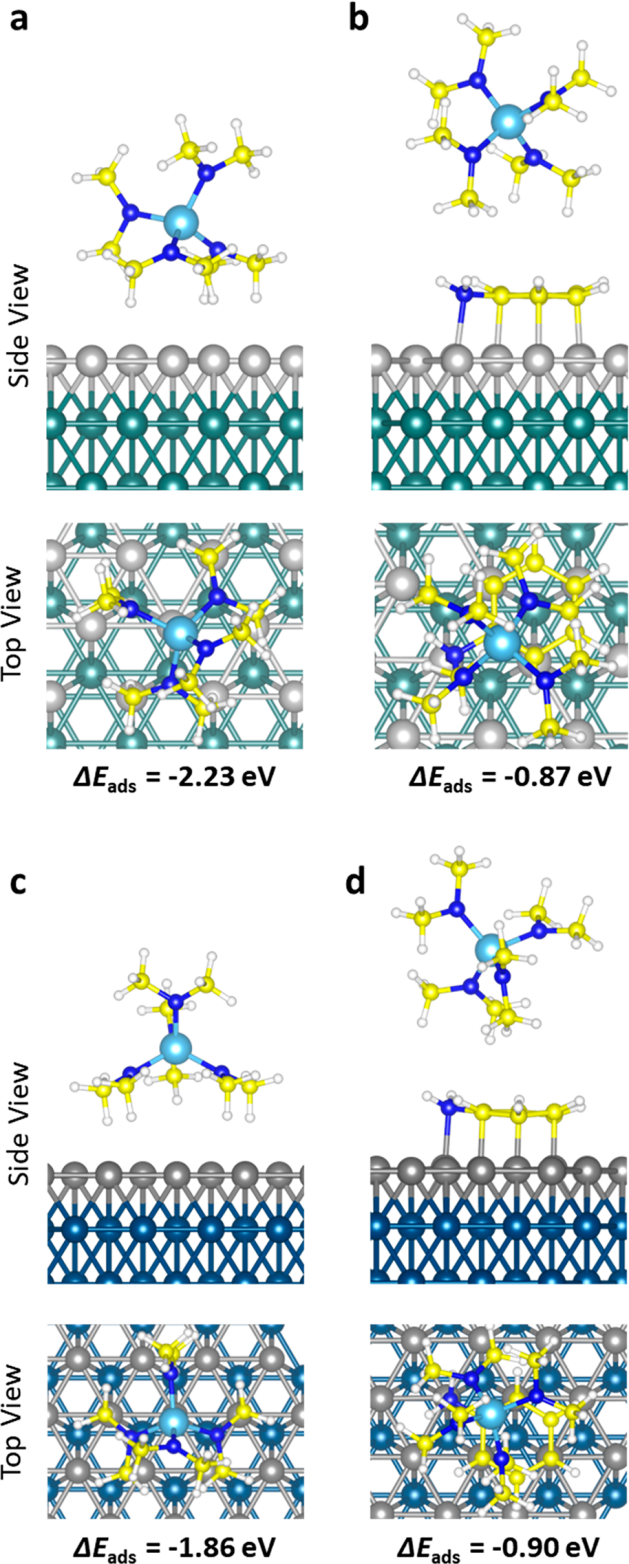
DFT-optimized TDMAT adsorption configurations and energies on (a) bare Ru(0001), (b) Ru(0001) functionalized with aniline, (c) bare Co(0001), and (d) Co(0001) functionalized with aniline (the light blue spheres represent Ti atoms, and the top layer metals are represented in a different color for better top view visualization).

In addition to the selective adsorption of aniline, its ability to block TDMAT (Ti precursor in ALD of TiN) was studied using DFT. To this end, the adsorption of TDMAT was studied on bare and aniline-functionalized Ru and Co surfaces (see Figure 3). On bare surfaces, we found TDMAT adsorption energies of −2.23 and −1.86 eV, on Ru and Co, respectively. To study the interaction of the precursor with the adsorbed aniline molecule, TDMAT is placed above the aniline adsorbate optimized in previous steps (which were shown in Figure 2). The interaction of TDMAT with the vertical aniline configuration resulted in the transformation to the horizontal configuration. This change of the aniline adsorption configuration mainly occurs as a result of repulsion interactions between the inhibitor and precursor molecules and shows that TDMAT is not likely to adsorb on vertical aniline. This transition was possible because only one aniline molecule was present on the surface in the DFT calculation. Note that such a transition is sterically hindered on a surface saturated with aniline molecules in both configurations. We calculated the adsorption energy of TDMAT on surfaces with aniline in the horizontal configuration as −0.87 and −0.90 eV on Ru and Co, respectively. In both cases, TDMAT is physisorbed on aniline at a distance of 2.6 Å. The lower adsorption energy of TDMAT on the aniline-functionalized surface compared to the bare surface confirms that the aniline inhibitor significantly reduces the interaction of TDMAT with the non-growth area.

In our previous work, introduction of the Si precursor bis(diethylamino)silane (BDEAS) on the Hacac-functionalized Al_2_O_3_ surface resulted in an adsorption energy of −0.81 eV on Hacac in monodentate configuration.^32^ In that case, we observed the formation of a H bond between the free OH group from adsorbed Hacac and the amino ligand in BDEAS, for some orientations of the BDEAS molecule. This interaction was described as the starting point for the displacement of the Hacac inhibitor from the surface by the precursor, which was experimentally observed. Interestingly, in the current analysis, the TDMAT adsorption energy on aniline is similar to that of BDEAS on Hacac. However, unlike the previous case, we do not observe such a H bond that could serve as a pathway for displacement. Although the thermodynamics of the aniline–TDMAT interaction resembles the Hacac–BDEAS case, the displacement kinetics are expected to be different for these two cases, considering the significantly higher selectivity observed for aniline in our experimental work.^28^

**Figure 4:**
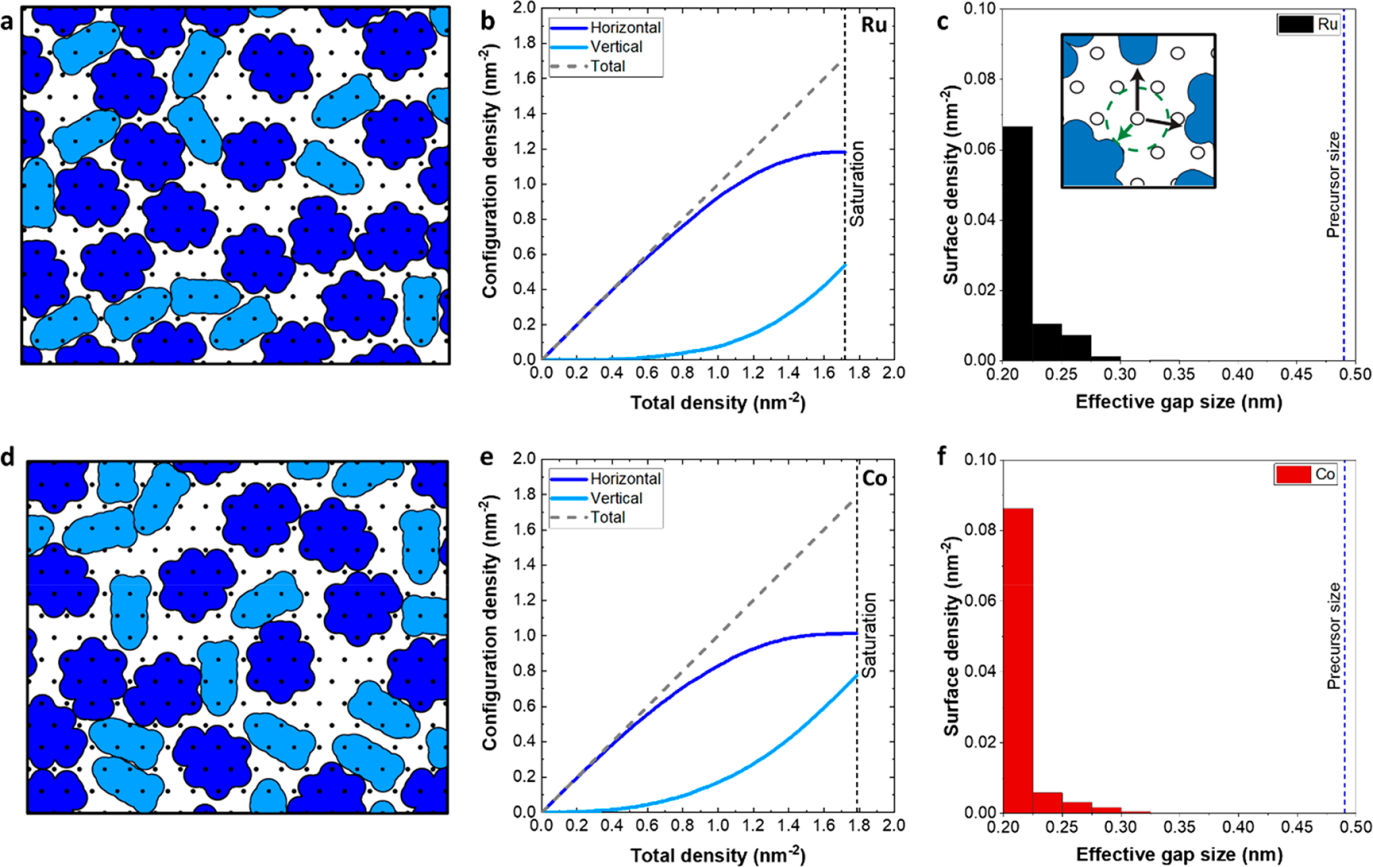
RSA simulation results for aniline adsorption on (a–c) Ru(0001) and (d–f) Co(0001) surfaces: (a and d) visual simulation output, (b and e) surface aniline configuration densities as a function of the total aniline density on the surface, and (c and f) distribution of effective gap sizes after aniline adsorption. The blue dashed line at 0.49 nm represents the size of the TDMAT precursor. The inset in panel c shows the procedure of determining the gap sizes by measuring the minimum distance between an unoccupied site and the nearest adsorbed inhibitor molecules (i.e., the radius of the largest circular molecule that can adsorb on the unoccupied site).

An effective small-molecule inhibitor should have a high enough surface coverage to be able to block the adsorption of precursor molecules. DFT-optimized aniline geometries on Ru and Co surface slabs, shown in panels a and b of Figure 2, were used to determine the 2D footprints (see Figure S2 of the Supporting Information) of the aniline molecule on a hexagonal grid representing the Ru and Co non-growth areas. Sample RSA outputs showing the packing of aniline molecules on zoomed in views of the Ru and Co non-growth area models are given in panels a and d of Figure 4. Our RSA algorithm starts with the selection of a random surface site and tests the adsorption of the energetically most favorable, horizontal, configurations of aniline for each iteration of the RSA simulations. When the horizontal configuration could not fit, adsorption of the vertical configuration was tested on the same adsorption site. Therefore, the aniline adsorption on both surfaces is initially dominated by the most stable horizontal aniline configurations. With increasing aniline exposure, we see a transition from the thermodynamically most favorable configuration to the sterically most favorable (i.e. the vertical) configuration. The change of the surface density of the specific adsorption configurations as a function of total aniline density on the surface is plotted in panels b and e of Figure 4. At saturation represented by black dashed lines (1.72 molecules/nm^2^) in Figure 4b, 69 ± 1% of the aniline molecules are present in the horizontal configuration on the Ru surface. The same analysis on the Co surface (Figure 4e) showed that 57 ± 1% of adsorbed aniline molecules are in the horizontal configuration, whereas the saturation surface density is 1.79 molecules/nm^2^. With the contribution of both adsorption configurations, 60 ± 1 and 59 ± 1% of the surface area is covered with aniline molecules on Ru and Co, respectively. Considering that both horizontal and vertical configurations are present on the surface in considerable quantities, both adsorption configurations should play a significant role in the precursor blocking observed in our experimental work.^28^

The RSA simulation results were also analyzed in terms of the sizes of the gaps in between adsorbed inhibitor molecules after surface functionalization with aniline. The distribution of the effective gap sizes on the non-growth area can be used to estimate whether a precursor molecule can potentially adsorb, as a measure for the selectivity.^31^ Gap size distribution analysis for the Ru and Co surfaces functionalized with aniline molecules indicates that the gaps on both surfaces are mainly concentrated below 0.35 nm, as reported in panels c and f of Figure 4. Considering the size of a 2D-projected TDMAT precursor molecule (∼0.49 nm, also shown as a blue dashed line in panels c and f of Figure 4), our results suggest that the inhibition layer is sufficiently densely packed such that there are no surface sites available that can accommodate the adsorption of precursor molecules. These results support that aniline can effectively shield the adsorption sites on Ru and Co non-growth areas and block the adsorption of the incoming precursor molecules.

**Figure 5:**
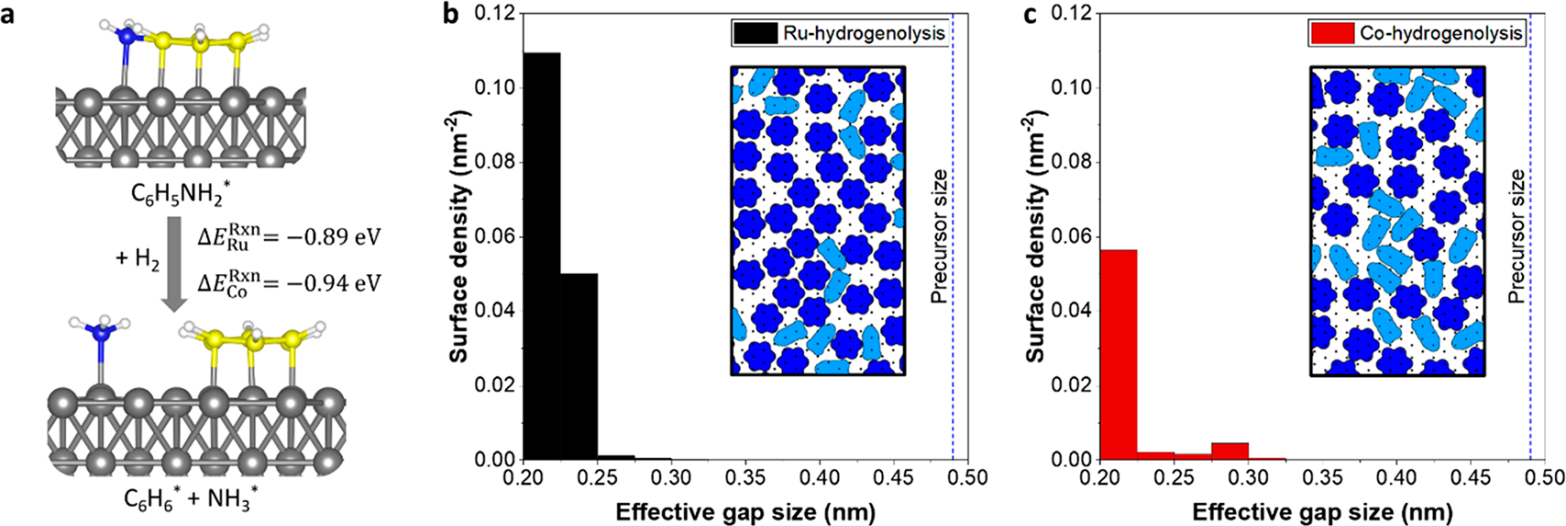
(a) Aniline hydrogenolysis on metal (Ru and Co) surfaces and (b and c) effective gap distributions on Ru and Co model surfaces after hydrogenolysis. The blue dashed line at 0.49 nm represents the size the TDMAT precursor. Insets show sample sections from the visual RSA outputs.

Literature on Pt, Ni, and Mo surfaces shows that, when hydrogen is present, aniline can undergo hydrogenolysis and dissociate into benzene and ammonia on the surface.^50−52^ Given that the co-reactant in the area-selective TiN ALD processes is an Ar–H_2_ plasma,^28^ it is reasonable to assume that hydrogen is also available during the aniline dose. Because Ru and Co are well-known catalysts, such a reaction can occur on the non-growth areas used in this work. Thus, we also consider the effect of hydrogenolysis on the inhibition process. Our DFT results show that the hydrogenolysis of adsorbed aniline is thermodynamically favorable on both metal surfaces. As represented in Figure 5a, this process has reaction energies of −0.89 and −0.94 eV on Ru and Co surfaces, respectively. Ammonia and benzene are formed on the metal surfaces as a result of the hydrogenolysis reaction. The formed ammonia species has low adsorption energy (−1.01 eV on Ru and −0.65 eV on Co) and can potentially desorb from the surface under the experimental conditions. Similar to aniline, the formed benzene molecules have strong adsorption energies of −2.88 and −1.87 eV on Ru and Co, respectively, and, therefore, do not easily desorb from the non-growth area.

A new set of RSA simulations was performed by assuming the hydrogenolysis of all incoming aniline molecules that adsorb in the horizontal configuration. According to these simulations, the density of adsorbed inhibitor molecules is increased on Ru from 1.72 to 1.99 nm^–2^ and on Co from 1.79 to 1.86 nm^–2^. At saturation, 68% of the adsorbed molecules are present as benzene on the Ru surface, while the rest of the adsorbates were aniline in vertical configuration. For the Co surface, the benzene fraction was calculated as 57%. As a result, the fraction of the surface area covered is increased on the Ru non-growth area from 60 ± 1 to 63 ± 1% and non-significantly changed from 59 ± 1 to 57 ± 1% on the Co non-growth area. The improvement in the surface saturation density can be explained by the adsorption of more inhibitor molecules on the newly available surface sites after ammonia byproduct molecules leave the surface. As seen from panels b and c of Figure 5, the density of the large gaps on the aniline-functionalized surface decreases after hydrogenolysis. RSA results, thereby, imply that hydrogenolysis is beneficial for precursor blocking. Furthermore, the elimination of the amine groups by hydrogenolysis of aniline can further help the precursor blocking by making the inhibitor layer more chemically inert. Hence, hydrogenolysis of adsorbed aniline molecules can contribute to the precursor blocking on both non-growth areas.

## Conclusion

In this work, on the basis of experimental results for the small-molecule inhibitor aniline, we computationally investigated how SMIs can effectively block precursor adsorption on the non-growth area during area-selective ALD. As the first requirement for ASD, aniline strongly adsorbs on the non-growth area, whereas its adsorption on the growth area is weak. From our DFT calculations for aniline and prior results for Hacac, it is concluded that all adsorption configurations of the inhibitor molecule should strongly adsorb on the non-growth area to obtain a stable inhibition layer. The case of aniline demonstrates that the presence of multiple stable binding configurations also results in a dense inhibitor packing, which helps the blocking of precursor adsorption on the non-growth area. As the surface starts to saturate, the energetically most favorable aniline configuration cannot adsorb on the remaining unoccupied sites. In that case, the sterically more favorable configuration can still adsorb to the surface and enhance the surface inhibitor density. The catalytic properties of the non-growth area can also contribute to precursor blocking performance. The hydrogenolysis of the adsorbed aniline molecule is found to be thermodynamically favorable on the studied metallic non-growth areas. Such catalytic reactions on the non-growth area can enhance the inhibitor blocking on the non-growth area by increasing the fraction of the surface area covered and/or decreasing the number of available gaps on the surface. In addition, the removal of the reactive amine groups can eliminate the possible interactions with the precursors.

Taken together, to effectively block precursor adsorption, an inhibitor should satisfy three main requirements: (i) all possible adsorption configurations of an inhibitor molecule should strongly and selectively adsorb on the non-growth area, (ii) precursor molecules should not be able to adsorb on or react with inhibitor molecules, and (iii) the (mixture of) adsorption configuration(s) of the inhibitor should result in a sufficiently high surface coverage with no gaps available for adsorption of the incoming precursor. In addition to these, if the inhibitor molecule reacts on the surface, the products should satisfy these requirements to achieve effective blocking of an incoming precursor molecule.

## Supporting Information Available

RSA algorithm (Figure S1), determination of 2D footprints of inhibitor molecules on the substrate model (Figure S2), and illustration showing how the effective gap size is determined (Figure S3) (PDF)
