# Supplementary material for: Computational Investigation of Precursor Blocking during Area-Selective Atomic Layer Deposition Using Aniline as a Small-Molecule Inhibitor
Source: Langmuir. 2023 Mar 15;39(12):4265–73. doi: 10.1021/acs.langmuir.2c03214 (PMC10061919; doi:10.1021/acs.langmuir.2c03214)
Supplement: Supplementary file 1 — la2c03214_si_001.pdf [file la2c03214_si_001.pdf]

Supporting Information

Computational investigation of precursor blocking during area-selective atomic layer deposition using aniline as a small molecule inhibitor

I. Tezsevin,[a] J. F. W. Maas,[a] M. J. M. Merkx,[a] R. Lengers,[a] W. M. M. Kessels,[a] T. E. Sandoval,[b] A. J. M. Mackus[a]

[a] *Department of Applied Physics, Eindhoven University of Technology, P.O. Box 513, 5600 MB Eindhoven, The Netherlands*

[b] *Department of Chemical and Environmental Engineering, Universidad Técnica Federico Santa Mariá, Santiago 2340000, Chile.*

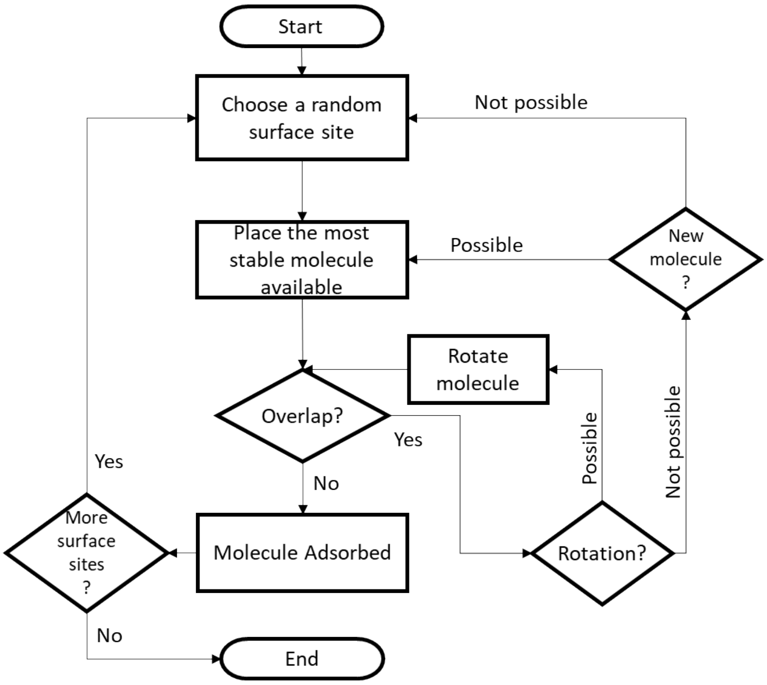

Figure S1. Algorithm followed in random sequential adsorption simulations.

In this algorithm, every surface site can be chosen only once in a random order. The energetically most stable configuration (in this case aniline in horizontal configuration) is attempted to adsorb first. A molecule can only adsorb if there is no physical overlap with other molecules on the surface. After adsorption, molecules were frozen in that position until the end of the simulation. If overlap with another molecule was detected, the molecule was rotated and attempted to be placed again. The horizontal aniline configurations were rotated with rotation steps of 60° where the vertical aniline configurations were first rotated with 60° and then 1°. After all possible random rotations were tried, the next most stable configuration was attempted to adsorb on the same site. If no more configurations were available, the surface site was flagged, and a new site was chosen. The simulations were terminated after all adsorption sites on the surface were tested. When the reactive adsorption mode is activated, aniline molecule adsorbed in horizontal configuration was immediately converted to benzene. The sticking probability was taken as unity in these simulations. The cooperative effects, diffusion, or desorption of the adsorbates were not taken into account in the RSA model development. All RSA results reported in this study were averaged over 20 simulations for the same process. Please see *J. Vac. Sci. Technol. A* 2022, *40* (6), 062409 for more information on the RSA methodology.

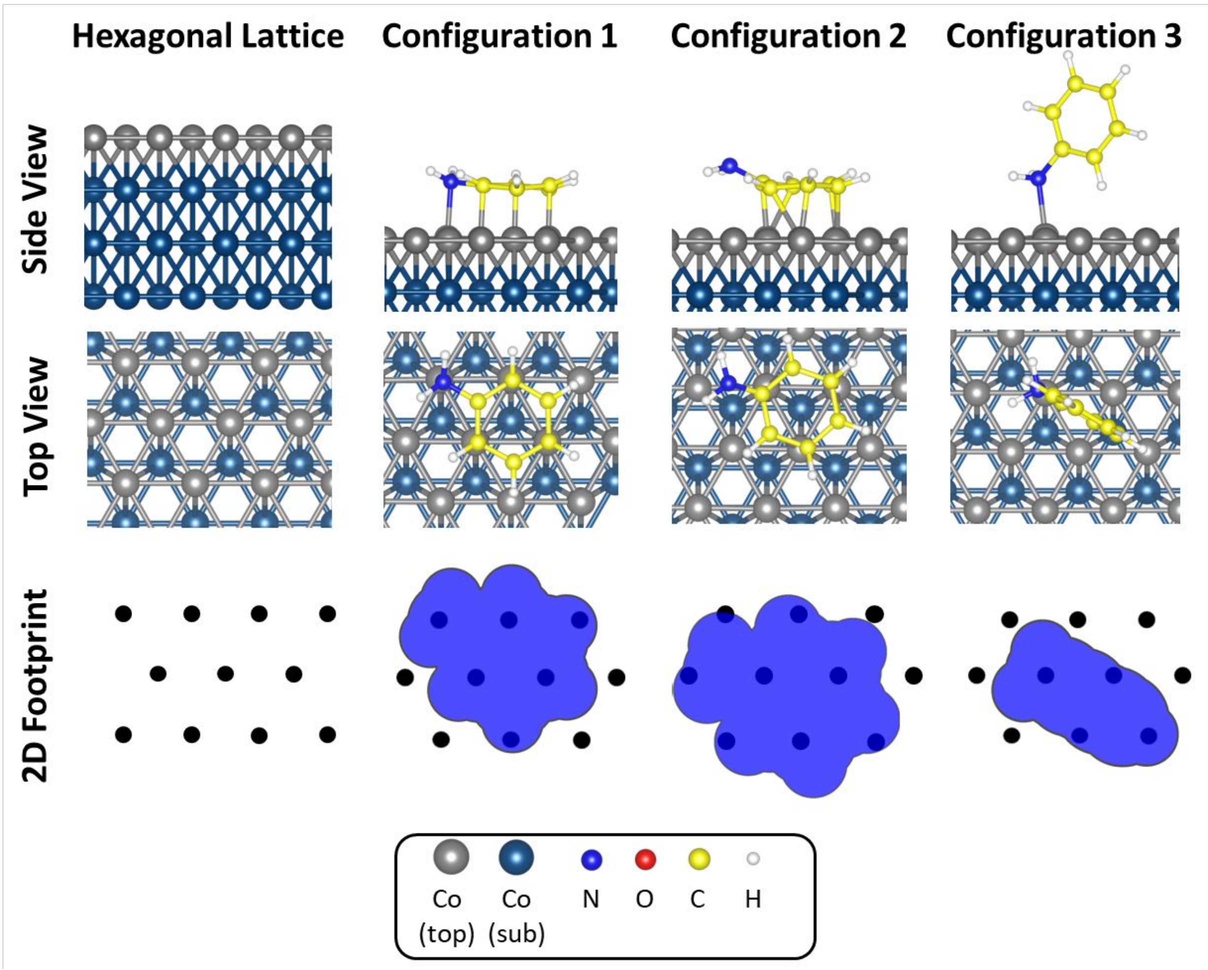

Figure S2. 2-dimensional footprints of aniline molecules used in the random sequential adsorption simulations on Co. The top view of the designated binding configuration is used to project the positions of the individual atoms creating the aniline molecules on x-y plane. Using the obtained coordinates and the van der Waals radius of the specific elements, the boundaries of the 2D footprint are determined.

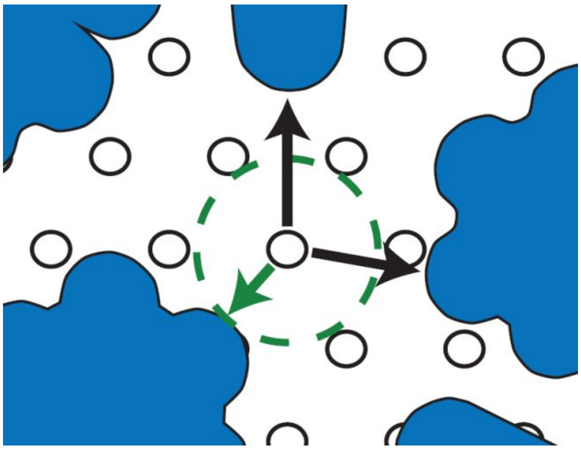

Figure S3. Illustration showing how the effective gap size is determined. For each surface site that is not occupied (or sterically not covered), the distance to each neighboring adsorbed inhibitor is determined. The smallest of these distances (indicated by the green arrows) is taken as the effective gap size. The effective gap size describes the largest circularly shaped molecule (in 2D) that could fit on the surface site without steric overlap with the surrounding adsorbed inhibitor molecules. (figure adopted from: Merkx, M. J. M. (2022). Area-selective atomic layer deposition using small molecule inhibitors. [Phd Thesis (Research TU/e / Graduation TU/e), Applied Physics]. Eindhoven University of Technology). Also see *J. Vac. Sci. Technol. A* **2022**, *40* (6), 062409 for more information on RSA.
